# A hydrophilic microenvironment in the substrate-translocating groove of the YidC membrane insertase is essential for enzyme function

**DOI:** 10.1016/j.jbc.2022.101690

**Published:** 2022-02-09

**Authors:** Yuanyuan Chen, Marcos Sotomayor, Sara Capponi, Balasubramani Hariharan, Indra D. Sahu, Maximilian Haase, Gary A. Lorigan, Andreas Kuhn, Stephen H. White, Ross E. Dalbey

**Affiliations:** 1 Department of Chemistry and Biochemistry, The Ohio State University, Columbus, Ohio, USA; 2 Department of Industrial and Applied Genomics, IBM AI and Cognitive Software Organization, IBM Almaden Research Center, San Jose, California, USA; 3 NSF Center for Cellular Construction, University of California in San Francisco, San Francisco, California, USA; 4 Department of Chemistry and Biochemistry, Miami University, Oxford, Ohio, USA; 5 Natural Science Division, Campbellsville University, Campbellsville, Kentucky, USA; 6 Institute of Microbiology and Molecular Biology, University of Hohenheim, Stuttgart, Germany; 7 Department of Physiology and Biophysics, University of California, Irvine, California, USA

**Keywords:** YidC, *S. mutans*, molecular dynamics, membrane biogenesis, membrane transport, DDM, n-Dodecyl-beta-D-Maltoside, EPR, electron paramagnetic resonance, ER, endoplasmic reticulum, Mal-PEG, methoxypolyethylene glycol maleimide, MTSL, (1-Oxyl-2,2,5,5-tetramethyl-Δ3-pyrroline-3-methyl) methanethiosulfonate, NiEDDA, nickel(II) ethylenediaminediacetate, PC, Procoat, PK, proteinase K, TM, transmembrane

## Abstract

The YidC family of proteins are membrane insertases that catalyze the translocation of the periplasmic domain of membrane proteins *via* a hydrophilic groove located within the inner leaflet of the membrane. All homologs have a strictly conserved, positively charged residue in the center of this groove. In *Bacillus subtilis*, the positively charged residue has been proposed to be essential for interacting with negatively charged residues of the substrate, supporting a hypothesis that YidC catalyzes insertion *via* an early-step electrostatic attraction mechanism. Here, we provide data suggesting that the positively charged residue is important not for its charge but for increasing the hydrophilicity of the groove. We found that the positively charged residue is dispensable for *Escherichia coli* YidC function when an adjacent residue at position 517 was hydrophilic or aromatic, but was essential when the adjacent residue was apolar. Additionally, solvent accessibility studies support the idea that the conserved positively charged residue functions to keep the top and middle of the groove sufficiently hydrated. Moreover, we demonstrate that both the *E. coli* and *Streptococcus mutans* YidC homologs are functional when the strictly conserved arginine is replaced with a negatively charged residue, provided proper stabilization from neighboring residues. These combined results show that the positively charged residue functions to maintain a hydrophilic microenvironment in the groove necessary for the insertase activity, rather than to form electrostatic interactions with the substrates.

The YidC/Oxa1/Alb3 proteins are found in bacteria, mitochondria, and chloroplast where they play a pivotal role in membrane protein biogenesis (1, 2). More recently, homologs have also been found in the eukaryotic endoplasmic reticulum (ER) membrane (3, 4, 5). This includes the Get1, EMC3, and TMCO1 proteins that function within large membrane complexes that play a key role in ER membrane protein biogenesis (5). In bacteria, YidC can function on its own (6, 7, 8, 9, 10, 11) or in concert with the Sec system to insert proteins into the cytoplasmic membrane (12, 13, 14). YidC helps facilitate the removal of transmembrane (TM) proteins from the Sec channel (15) and assists in the formation of α-helical bundles of membrane proteins (16, 17).

In 2014, the structures of YidC from *Bacillus halodurans* (18) and *Escherichia coli* (19) were solved at high resolution using X-ray crystallography. These structures showed that the YidC protein possesses a five TM segment core domain (TM2-TM6 in the case of the *E. coli* YidC) with an unusual hydrophilic cavity located within the inner leaflet of the membrane that is accessible from the cytoplasm and lipid bilayer, but not from the periplasm. In addition, YidC possesses a conserved coiled-coil region in the cytoplasm comprised of two α-helices CH1 and CH2 that most likely is involved in substrate binding of YidC substrates such as Pf3 coat (20), as well as binding of SRP and the SRP receptor FtsY that targets ribosome nascent chains to YidC (21). Interestingly, CH1 forms a continuous helix with the first TM region of the core domain (TM2 of *E. coli* YidC), which is kinked by two conserved prolines, one in TM1 of the core (TM2 of *E. coli* YidC) and one in front of CH1. These prolines along with the disordered loop (Pro-Leu-Gly-Gly-Cys-Phe-Pro in *E. coli* YidC) connecting CH2 to the second TM core segment (TM3 of *E. coli* YidC) likely function to move the substrate from the coiled-coil domain into the hydrophilic groove.

The *E. coli* YidC residues that have been determined as contacting the TM segment of substrates during insertion were found to cluster on the same face of TM3 and TM5 that forms a ‘greasy slide’ (1, 22), while the hydrophilic region of the substrate was incorporated transiently into the hydrophilic groove prior to translocation across the membrane (23). The cavity contains a strictly conserved positively charged residue that is essential in *Bacillus subtilis* (18). This residue has been proposed to participate in an electrostatic step to translocate the negatively charged N-terminal tail region of the MifM protein across the membrane (18). However, the conserved positive charge is not essential for the insertase activity of the *E. coli* YidC or an *Arabidopsis thaliana* Alb3 derivative (24).

A molecular dynamics (MD) simulation of *E. coli* YidC embedded in a palmitoyloleoyl-phosphatidylethanolamine:palmitoyloleoyl-phosphatidylglycerol (POPE:POPG) phospholipid bilayer showed the protein to be more compact than the crystal structure and reveals significant thinning in the vicinity of the protein (25). The thinning of the membrane bilayer and the presence of the hydrophilic groove reduces the energy cost for translocation, which is a new paradigm in biology (26). The strictly conserved arginine 366 in the hydrophilic groove of the *E. coli* YidC is in proximity to tyrosine 516 and 517 at the top of the groove (25) (Fig. 1) and is likely hydrogen bonded *via* water molecules. These tyrosine residues are part of an aromatic cluster at the interface between the aqueous environment in the groove and the embedded hydrophobic portion of YidC in the outer leaflet of the membrane.

**Figure 1:**
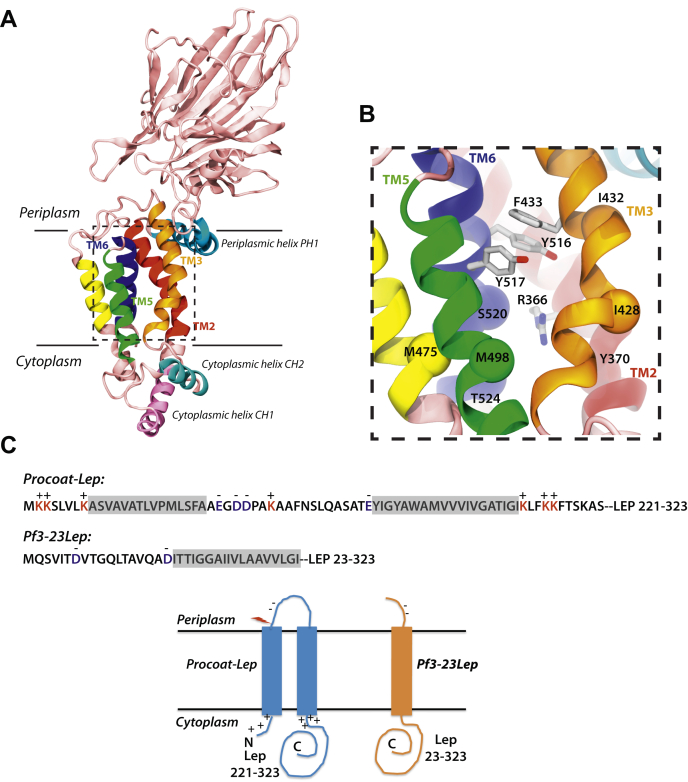
**Structure of the *Escherichia coli* YidC and domain architecture of substrates.***A*, structure of the *Escherichia coli* YidC depicting its transmembrane and cytoplasmic helices. *B*, a closeup view of the hydrophilic groove (PDB 3WVF) (25). Residues studied in this paper are indicated, including the conserved positively charged R366, as well as the tyrosines and groove residues. Side chains are shown as *stick* for R366 and the aromatic residues mutated to study the requirement of the conserved arginine, while Cα atoms are shown as *spheres* for groove residues studied in Cys-alkylation assay. *C*, YidC substrates PC-Lep and Pf3-23Lep used in this study (see Experimental procedures for details) and their membrane topologies. The *redarrow* in PC-Lep depicts the cleavage site for signal peptidase. PC, procoat.

To gain further insights into the role of R366 in the YidC insertase activity, we investigated the function of this conserved positively charged residue in the hydrophilic groove of the *E. coli* YidC and *Streptococcus mutans* YidC2. We found that the positive charge (R366) is important for the *E. coli* YidC when a nearby tyrosine at position 517 is changed to an amino acid with an apolar side chain. However, it is not essential when the tyrosine is changed to a hydrophilic or aromatic amino acid. Similar results were also found with *S. mutans* YidC2. Solvent accessibility assays show that the arginine is required to keep the upper part of the groove solvent accessible when Y517 is replaced with an apolar residue. Strikingly, both *E. coli* YidC and *S. mutans* YidC2 can function in membrane protein insertion when the arginine is replaced with a negatively charged glutamic acid residue, provided that a suppressor mutation is also present, which most likely stabilizes the groove region. Taken together, these results support the hypothesis that the positively charged residue is employed to keep the groove hydrophilic and water exposed rather than to attract the negative charge region of the substrate to be translocated.

## Results

### The conserved positively charged residue R366 of *E. coli* YidC becomes functionally indispensable by mutation of tyrosine 517

**Figure 2:**
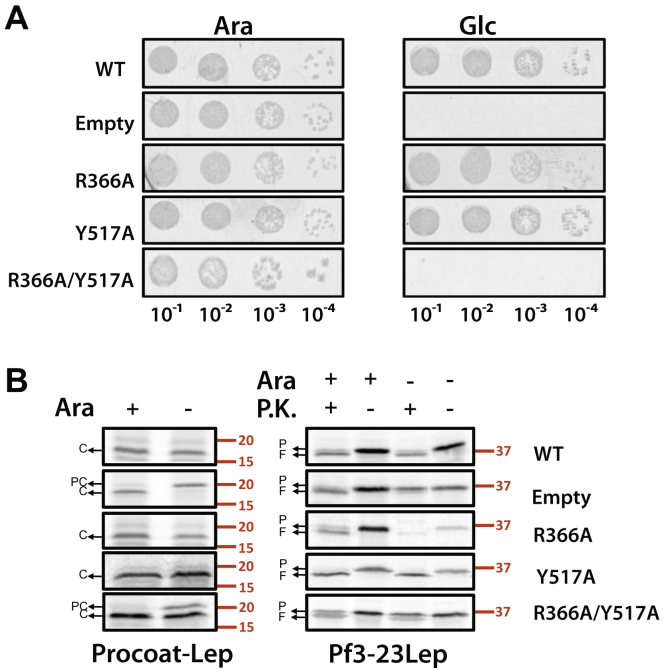
**The conserved R366 residue in *Escherichia coli* YidC becomes essential in the presence of the Y517A mutation.***A*, complementation assay to test the effect of mutating R366 and Y517 on *Escherichia coli* YidC function. pACYC184 encoding R366A, Y517A, and R366A/Y517A YidC mutants were constructed using site-directed mutagenesis and transformed into YidC depletion strain JS7131. After the depletion of endogenous YidC for 3 h, serial dilutions of the cells were spotted and incubated under YidC expression conditions (*left panels*) and depletion conditions (*right panels*), respectively (37 °C). Plasmid encoding WT YidC and empty plasmid were also expressed, as positive and negative controls. *B*, same *E. coli* YidC mutants were tested for their ability to insert PC-Lep (*left panel*) and Pf3-23Lep (*right panel*). JS7131 harboring pACYC184 encoding WT YidC, empty plasmid, or YidC mutants were cotransformed with pMS119 encoding PC-Lep or Pf3-23Lep. The cells were grown under YidC expression conditions (ara+) or depletion conditions (ara−) for 4 h (37 °C). Expression of the substrates was induced by 1 mM IPTG for 5 min and labeled with [^35^S]-methionine for 1 min. Signal peptide processing assay was used to assess the insertion of PC-Lep (*left panel*), and the insertion of Pf3-23Lep was examined by protease mapping (*right panel*), both as described in “Experimental procedures”. *Red numbers* indicate molecular weight marker values in kDa. C, Coat-Lep; PC, Procoat-Lep.

Previous structural (19) and MD simulation studies (25) revealed that the aromatic residues at 516 and 517 in the *E. coli* YidC are located above arginine 366 at the top of the hydrophilic groove (Fig. 1*B*). Our first question concerned the necessity of these two aromatic residues. Y516, Y517, or both Y516 and Y517 were mutated to alanines. The activities of these mutants were determined utilizing the YidC depletion strain JS7131, with the endogenous *yidC* knocked out and a new copy of *yidC* gene under the control of the *araBAD* promoter introduced at the lambda attachment site (6). The JS7131 cells harboring WT YidC or the YidC mutants were analyzed by serial dilution on a LB glucose or arabinose plate at 37 °C. We found that the Ala substitution of Y517 (Figs. 2*A* and S1), Y516 (Fig. S1), or Y516/Y517 (Fig. S1) complemented the YidC depletion strain. However, when R366 was mutated to Ala or Cys, then Y517 was important for the activity but not Y516. Ala or Cys substitutions of R366 combined with Y517A did not complement the YidC depletion strain (Figs. 2*A* and S1), while the single Ala substitutions of R366 (Fig. 2*A*) and double R366A/Y516C did complement (Fig. S1). Next, we examined directly the activity of the mutants by testing the membrane insertion of two YidC-dependent proteins. First, we analyzed the model protein procoat-Lep (PC) (Fig. 1*C*). Procoat-Lep corresponds to PC with its C-terminal cytoplasmic region extended by 101 amino acids of the periplasmic domain of leader peptidase (Lep) (9). If PC-Lep inserts across the membrane, it is cleaved by signal peptidase 1 and converted to coat-Lep (C). The R366A and Y517A YidC single mutants were fully active in inserting PC-Lep while the double R366A/Y517A mutant was inhibited leaving PC partially uncleaved (Fig. 2*B*, -Ara condition). Similar results were seen with the Pf3-23Lep substrate, which has the Lep residues 23 to 323 fused after the TM segment of Pf3 coat and an arginine introduced after the TM of Pf3-23Lep to prevent translocation of the Lep C-terminal domain (24) (Fig. 1*C*). When Pf3-Lep (P) inserts across the membrane, proteinase K (PK) digests the N-tail of Pf3-Lep and converts it to a smaller fragment (F). Figure 2*B* shows that Pf3-23Lep was inserted efficiently with either the R366A or Y517A single mutant, while insertion was blocked with the R366A/Y517A double mutant (Fig. 2*B*, - Ara condition). It is worth noting that unlike Pf3-23Lep, which showed almost complete loss of insertion with the double mutant, PC-Lep was only partially inhibited. This has also been observed previously (24), possibly due to the different insertion mechanisms employed by the two substrates.

For all the mutants described here, the protein was stably expressed as determined by Western blotting (Fig. S5). The requirement of the positive charge was confirmed by substituting R366 with neutral (Ala, Cys, Asn), positively charged (Lys), or negatively charged (Asp) residues in the presence of Y517A mutation. As shown in Fig. S1, only the lysine mutant, R366K/Y517A, is active as it complemented the YidC depletion strain. The combined results show that we were able to make the arginine 366 in *E. coli* YidC essential by mutating the nearby tyrosine 517 residue.

### Functional importance of the *E. coli* YidC conserved positively charged residue depends on the identity of residue 517

**Figure 3:**
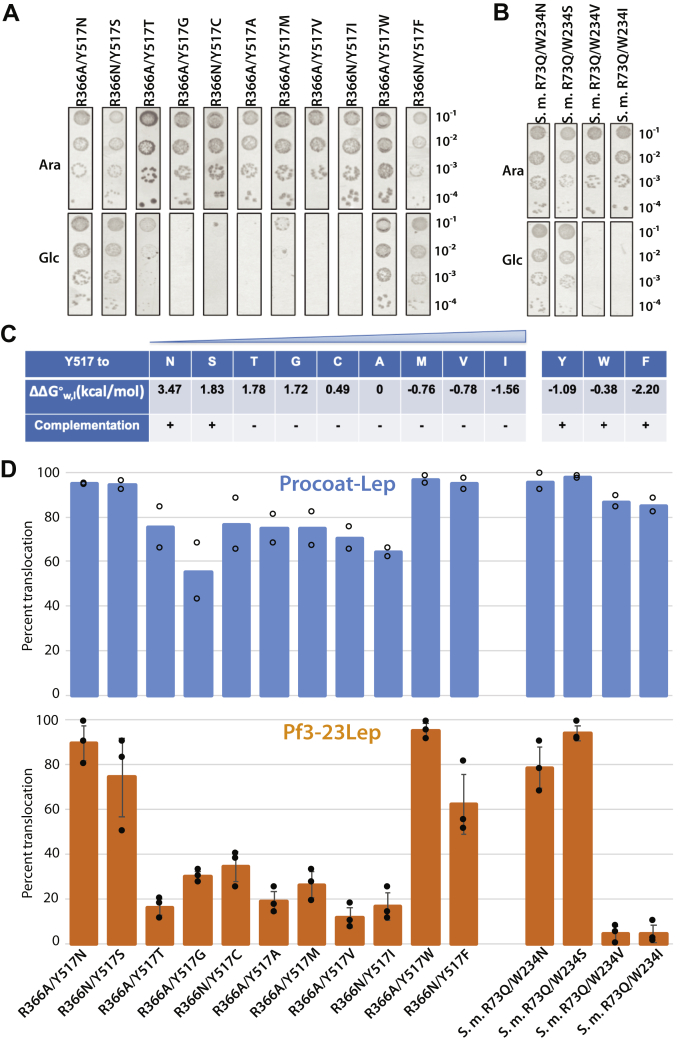
**The polarity at the dome of the YidC hydrophilic groove determines the necessity of the conserved positive charge.***A*, complementation assay to examine the importance of R366 (by mutating R366 to Ala or Asn) for *Escherichia coli* YidC when Y517 was substituted with Asn, Ser, Gly, Thr, Ala, Cys, Val, Ile, Met, Trp, or Phe. YidC depletion strain JS7131 was transformed with pACYC184 encoding these double mutants and a spot test at 37 °C was performed to test complementation, as described in Figure 2*A*. Note the data for R366A/Y517A (see Fig. 2*A*) is included for comparison. *B*, complementation assay to examine the role of R73 (by substituting R73 with Q) of *Streptococcus mutans* YidC2 when W234 was mutated to Asn, Ser, Val, or Ile. Note that in *S. mutans* YidC2, W234 aligns with Y517 in *E. coli* YidC, while R73 is equivalent to R366. *C*, summary of complementation results for the *E. coli* YidC 517 mutants. The hydrophobicity panel shows the standard free energy in kcal/mol for each amino acid tested (42). The “+” indicates that the mutant complemented the YidC depletion strain, indicating the arginine is not essential. The “−” means it did not complement showing the arginine is required for function. *D*, *E. coli* YidC and *S. mutans* 247YidC2 mutants were tested for their ability to insert PC-Lep (*blue bars*) and Pf3-23Lep (*orange bars*). Plasmids pACYC184 encoding YidC or YidC2 mutants were cotransformed with pMS119 encoding PC-Lep or Pf3-23Lep into JS7131. After the expression of YidC substrates and labeling, the membrane insertion of PC-Lep and Pf3-23Lep was tested as described in Figure 2*B*. The results (one representative trial shown in Fig. S2) were quantified as previously described (24) and summarized in panel *D*. PC, procoat.

To address what characteristics of residue 517 determines whether R366 is required for YidC function, we substituted tyrosine 517 with an apolar, aromatic, or polar residue and tested whether YidC is functional when the positive charge (R366) is removed. Complementation studies revealed that the R366 neutral mutant (R366A or R366N) did not complement the YidC depletion strain when combined with an apolar substitution for Y517 (Y517G, Y517C, Y517A Y517V, or Y517I), while very poor complementation was observed for the Y517T and Y517M mutants (Fig. 3, *A* and *C*). In contrast, when the residue at position 517 had a hydrophilic (517N, 517S) or aromatic side chain (517W and 517F), we observed full complementation under YidC depletion conditions (Fig. 3, *A* and *C*). For the 366 residue, no difference was observed if there was a slightly hydrophobic alanine or a hydrophilic asparagine residue (data not shown). As a second test of YidC activity, we performed membrane insertion assays using PC-Lep and Pf3-23Lep (Fig. S2) and determined the amount of inserted protein by quantitation (Fig. 3*D*). Membrane insertion of PC-Lep and Pf3-23Lep was inhibited when the 517 tyrosine was changed to an apolar, nonaromatic amino acid, and arginine 366 was changed to either an Ala or Asn residue corroborating the complementation results (Fig. S2).

### The essential arginine R73 can be made dispensable by mutation of W234 in *S. mutans* YidC2

Previously, we found that the strictly conserved arginine R73 in *S. mutans* 247YidC2 (equivalent to R366 in *E. coli*) is essential as the protein was inactive when the R73 residue in the hydrophilic groove was substituted with a neutral or negatively charged residue (24). 247YidC2 consists of residues 1 to 247 of the *E. coli* YidC fused to residues 25 to 310 of *S. mutans* YidC2 containing the conserved arginine at position 73. Since the hydrophobicity at residue 517 can determine whether the R366 is essential or not in the *E. coli* YidC, we went on to test whether mutation of the W234 residue (corresponding to the Y517 residue in *E. coli*) in the *S. mutans* YidC2 could affect the requirement of R73 (corresponding to R366 in *E. coli*). Remarkably, when the W234 residue at the top of the groove of 247YidC2 was substituted with amino acids with hydrophilic sidechains (Asn or Ser), YidC2 could complement the YidC depletion strain even without the positively charged residue in the groove (R73Q), while it did not complement with an apolar amino acid at 234 (Val or Ile) (Fig. 3*B*). As expected, the membrane-insertion activities of the *S. mutans* R73Q/W234V and R73Q/W234I 247YidC2 mutants were inhibited for the PC-Lep substrate and was completely abolished for the Pf3-Lep substrate (Figs. S2 and 3*D*).

Taken together, these results reveal a pattern that applies to both *E. coli* YidC and *S. mutans* YidC2: the conserved positively charged residue is required for activity when there is an apolar residue at position 517 in the *E. coli* YidC or at position 234 in the *S. mutans* YidC2, but not when there is a hydrophilic residue at these sites.

### The conserved positively charged residue R366 keeps the *E. coli* YidC hydrophilic cavity hydrated when Y517 is substituted with an apolar residue

The results reported above show a clear correlation between the requirement of the conserved positive charge and the hydrophobicity near the top of the hydrophilic groove in both *E. coli* YidC and *S. mutans* YidC2. We hypothesize that the positively charged residue is required to maintain a hydrophilic environment in the groove.

**Figure 4:**
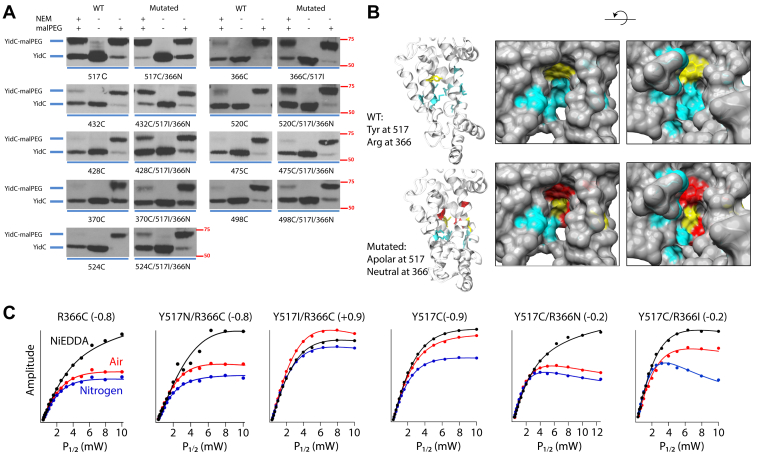
**The conserved positive charge R366 in *Escherichia coli* keeps the top part of the groove hydrated when Y517 is substituted with an apolar residue.***A*, cys-alkylation assay at 30 °C to examine the solvent accessibility of nine previously determined groove residues under WT conditions (lanes 1–3 in each panel) or mutated conditions (lanes 4–6). BL21 cells expressing the single Cys mutants were analyzed for modification by NEM using an indirect gel shift assay involving Mal-PEG, as described in “Experimental procedures”. Proteins were analyzed by SDS-PAGE, and YidC was detected by Western blotting using anti-6X His tag antibody (HRP). WT condition refers to the condition when Y517 and R366 were maintained, except for the cases when 517C and 366C were tested. Mutated condition refers to the condition when Y517 was substituted with an apolar residue (Ile or Cys), and R366 was mutated to a noncharged residue (Asn or Cys). *Red numbers* indicate molecular weight marker values in kDa. *B*, the solvent accessibility is summarized for the nine groove residues studied in panel *A* as previously described (25). *Cyan* means completely accessible to water; *yellow* represents partially solvent accessible; and *red codes* for completely inaccessible to water. Side and cytoplasmic views are shown. *C*, EPR power saturation assay at 22 °C (295K) to determine the water accessibility of residues 366 and 517 under WT conditions (R366C and Y517C) or mutated conditions (Y517N/R366C, Y517I/R366C, Y517C/R366N, and Y517C/R366I). The YidC mutants were overexpressed from BL21 cells, purified, spin-labeled with MTSL, reconstituted into DOPC liposome, and subjected to CW-EPR power saturation experiment as described in “Experimental procedures”. Water-soluble NiEDDA or insoluble air was applied as paramagnetic quencher, while diamagnetic nitrogen was used as a control. *P*_1/2_ values (the saturation parameter) were obtained from the saturation curves, and *Φ* values (the immersion depth parameter) were calculated using Equation 2 in “Experimental procedures”. DOPC, 1,2-dioleoyl-sn-glycero-3-phosphocholine; HRP, horseradish peroxidase; Mal-PEG, methoxypolyethylene glycol maleimide; NiEDDA, nickel(II) ethylenediaminediacetate.

To test this hypothesis, we examined the solvent accessibility of nine previously determined groove residues under WT and mutated conditions in the intact bacterial cells at 30 °C using a well-developed cysteine alkylation assay (25). Using this technique, each of the groove residues were mutated to single cysteine residues. If the cysteine is in a water-exposed environment, it will be reactive toward N-ethyl maleimide (NEM) because the cysteine thiol is deprotonated. If the cysteine is in a lipid-exposed environment, then the cysteine will not react with NEM because the thiol is protonated. To determine whether the YidC cysteine is modified by NEM, we treated the samples in a later step with methoxypolyethylene glycol maleimide (Mal-PEG), which leads to a 5 kD shift in the molecular weight of YidC, only if it had not reacted with NEM (lipid exposed). Therefore, a change in the position of YidC on the gel when NEM is added prior to the addition of Mal-PEG indicates exposure to lipid. If there is no shift with Mal-PEG when NEM is added, the Cys is solvent exposed. The WT condition starts with the Cys-less YidC (C423S) (Fig. 4*A*) and refers to the circumstance where Y517 and R366 are left unmutated except when their own solvent accessibility is studied (top panels). Under the mutated condition, Y517 is exchanged to an apolar isoleucine residue and R366 is substituted with a neutral asparagine amino acid simultaneously.

Figure 4*A* shows that the solvent exposure of Y517C is decreased considerably when the R366 is changed to a neutral asparagine. Similarly, R366C is much less water accessible when Y517 is mutated to apolar isoleucine residue. Likewise, the solvent accessibility is decreased for 432C when Y517 and R366 are substituted with an Ile and Asn, respectively. A less dramatic effect on water exposure is observed further down in the groove. With the Y517I and R366N mutations introduced, the solvent exposures of S520C and I428C residues are decreased, but to a lesser extent, than the cysteine mutants in the upper region of the groove. A negligible effect was observed when the water accessibility was examined toward the cytoplasmic side of the groove at positions M475, Y370, M498, and T524 when the Y517I and R366N mutations were made. A summary of the results is shown in Figure 4*B*, with the top panels representing the WT and the bottom panels the mutated construct. Cyan indicates residues that are completely water exposed, yellow moderate accessibility, and red residues that are not solvent accessible. The top panel shows that the groove is mostly aqueous under WT condition, while a cluster of red and yellow residues can be observed at the dome of the groove under mutated condition as revealed by the bottom panel, implying that the top part of the groove is not hydrated in these cases.

To confirm these results, we also performed electron paramagnetic resonance (EPR) power saturation studies to examine solvent accessibility of a spin label incorporated into the hydrophilic groove. His-tagged YidC mutants listed in Figure 4*C* were purified, spin-labeled with (1-Oxyl-2,2,5,5-tetramethyl-Δ3-pyrroline-3-methyl) methanethiosulfonate (MTSL), reconstituted into 1,2-dioleoyl-sn-glycero-3-phosphocholine liposomes, and subjected to EPR power saturation experiments at 22 °C as described in “Experimental procedures”. The saturation curves for each mutant in the presence of either nitrogen, oxygen, or nickel(II) ethylenediaminediacetate (NiEDDA) are shown in Figure 4*C*, from which the saturation parameters *P*_1/2_ were obtained and the immersion depth parameters *Φ* were calculated using Equation 2 in “Experimental procedures”.

As a control, a spin label was introduced at position 366 and determined to be solvent accessible as indicated by the highly negative *Φ* value (−0.8). When Y517 was substituted with a hydrophilic residue (Y517N), position 366 remained accessible (*Φ* = −0.8). However, the spin label at 366 was no longer water accessible (*Φ* = 0.9) when the apolar residue isoleucine was introduced at position 517 (Fig. 4*C*).

A spin label was also attached to the top of the hydrophilic groove (Y517C) and studied with different mutations at R366. Figure 4*C* shows that in all cases studied (Y517C, R366N/Y517C, R366I/Y517C), position 517 was accessible to solvent. However, a closer investigation of the *Φ* values suggested that when the positive charge was removed (R366N/Y517C or R366I/Y517C), 517 became much less solvent accessible (−0.2) than when it was present (−0.9), indicating that the positive charge at 366 contributes to keeping the groove hydrated.

Additionally, we carried out a series of comprehensive 200-ns long MD simulations of WT *E. coli* YidC and the Y517I/R366N mutant at ∼27 °C (300 K) embedded in a POPE:POPG (75:25) lipid bilayer. To evaluate the level of hydration at various locations within the groove, the number of water molecules within 6 Å of Cα atoms of various groove residues during the last 40 ns was counted (25) and normalized to the standard-state accessible surface areas A^o^ of the fully exposed residues of soluble proteins as previously reported (25). Fig. S3 shows that the water accessibility decreases surrounding residues 366, 520, 521, and 527 in the middle and bottom part of the groove for the construct Y517I/R366N compared to the WT YidC.

The combined results support our hypothesis that the positive charge at site 366 in *E. coli* is required to maintain a hydrophilic microenvironment in the groove when Y517 is substituted with an apolar residue. We note that 517C appears to be more solvent accessible in EPR studies compared to in alkylation studies, possibly due to the size or polarity of the MTSL spin label.

### The YidC hydrophilic groove can function with a negatively charged residue

**Figure 5:**
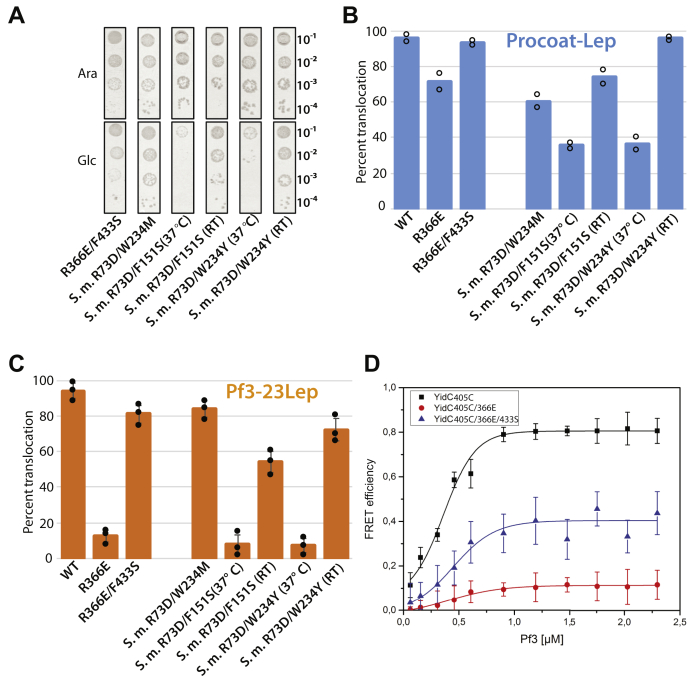
**YidC can function with a negatively charged residue substituting for the conserved positive charge in the groove.***A*, complementation assay to examine the activity of *Escherichia coli* YidC and *Streptococcus mutans* YidC2 mutants with a negative charge in the groove. The suppressor mutants F433S for *E. coli* YidC and W234M for *S. mutans* YidC2 were identified using mutator strain XL1-Red as described in “Experimental procedures”. F151 in *S. mutans* YidC2 aligns with *E. coli* YidC F433. All spot tests were performed at 37 °C unless indicated (RT). *B* and *C*, the insertase activity (results from one representative trial shown in Fig. S4) of *E. coli* YidC and *S. mutans* YidC2 negatively charged mutants were tested for the insertion of PC-Lep (*B*, *blue bar*) and Pf3-23Lep (*C*, *orange bar*) and performed as described in Figure 3*D*. JS7131 used in all insertion assays were grown at 37 °C unless specifically indicated (RT). *D*, Pf3 coat protein binding to *E. coli* YidC assayed by FRET. The Pf3 coat protein was labeled with the acceptor dye Atto647N at position 48 in the C-terminal tail. After a cysteine was introduced at position 405, the YidC mutants were expressed, purified, and labeled with the donor dye Atto520 at 405C in the cytoplasmic loop C1 and reconstituted in DOPC liposomes. The starting concentration of *E. coli* YidC was 0.3 μM, and the Pf3 coat was added in increasing amounts in 1 min steps. The fluorescence signal at the acceptor signal (ΔF_acceptor_) was plotted against the substrate concentration. YidC (*black*), YidC R366E (*red*), and the double mutant YidC R366E/F433S (*blue*) were analyzed. DOPC, 1,2-dioleoyl-sn-glycero-3-phosphocholine; PC, procoat.

**Figure 6:**
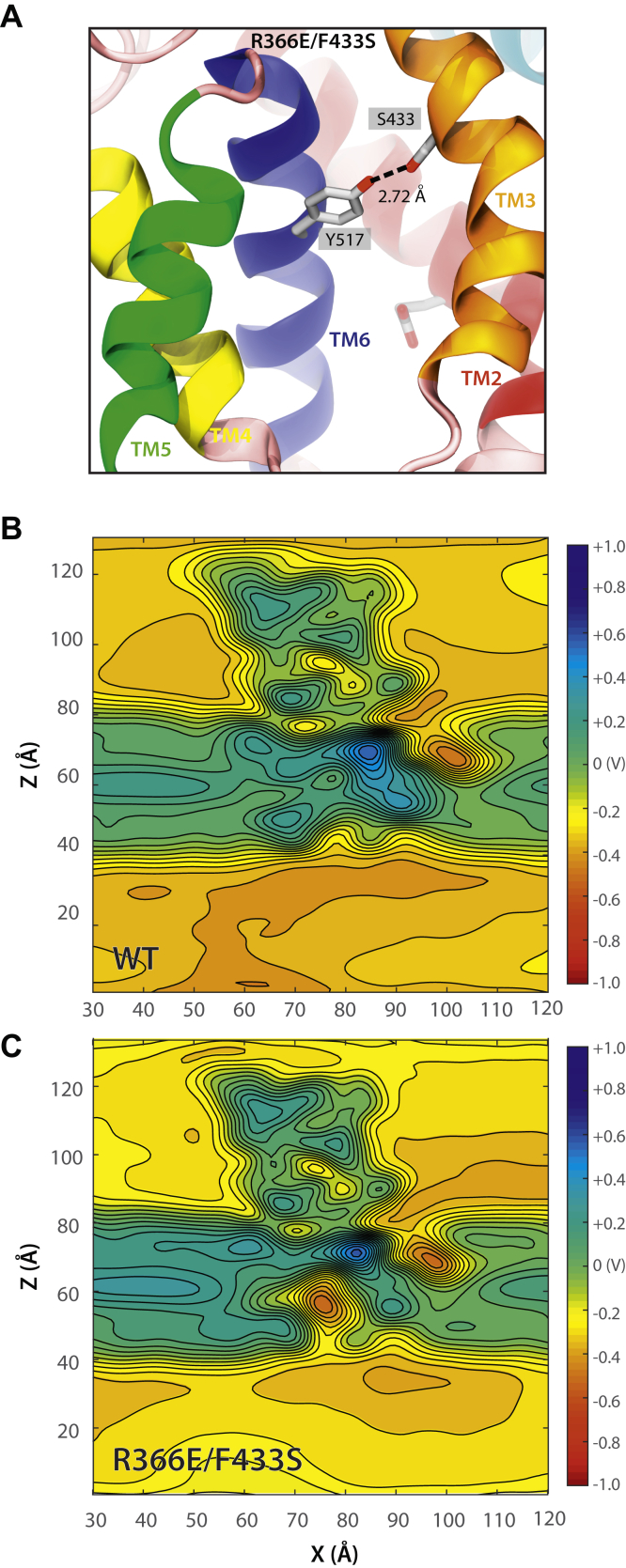
**The *Escherichia coli* YidC R366E/F433S protein with a negative charge in its groove is functional.***A*, snapshot of the equilibrated *Escherichia coli* YidC R366E/F433S system simulated in a hydrated POPE:POPG (75:25) bilayer (phosphopholipids and water are not shown; simulation S3b at ∼28 °C in Table 1). An interaction between the side chain of F433S (suppressor mutation) and the tyrosine residue at site 517 is observed during the simulation trajectory (Fig. S6). This interaction might stabilize YidC’s groove. *B* and *C*, two-dimensional contour plot of the averaged electrostatic potential computed during 100-ns long simulations of *E. coli* YidC at ∼28 °C with the protein backbone constrained (WT and R366E/F433S systems in *B* and *C*, respectively; simulations S1a and S3a in Table 1). The map corresponds to the electrostatic potential of a slice perpendicular to the membrane plane passing through the center of the protein near site 366. Twenty contour lines are drawn over the range of voltages. All values above or below the scale limits (color scale) are shown at the same level. The electrostatic potential for the R366E/F433S system is negative near the 366 site (*C*). POPE:POPG, palmitoyloleoyl-phosphatidylethanolamine:palmitoyloleoyl-phosphatidylglycerol.

If the arginine in the groove serves to make the groove sufficiently hydrated, then a negative charge should also function in this capacity. However, previous studies showed that the negative charge inactivated YidC (24). To determine if it is possible to obtain a functional *E. coli* YidC with a negative charged residue replacing the arginine at site 366, we searched for suppressor mutations of the inactive YidCR366E mutant. Intriguingly, we did obtain one suppressor mutation, R366E/F433S, after randomly mutagenizing YidC in the XL1 red mutator strain. This secondary mutation allowed the R366E YidC to complement the YidC depletion strain under glucose condition (Fig. 5*A*). The new serine side chain (F433S) is located above site 366 in the hydrophilic groove and may interact with the tyrosine 517 residue (see Fig. 6*A*). As expected, the R366E/F433S YidC mutant promotes membrane insertion of PC-Lep and of Pf3-23Lep (Figs. 5, *B* and *C* and S4). Further, in a reconstituted *in vitro* system, we examined the binding of R366E and R366E/F433S YidC to a fluorescently labeled Pf3 coat protein substrate (Fig. 5*D*). To fluorescently label YidC, a cysteine was introduced in the cytoplasmic loop C1 at position 405 in the cysteine-less WT, R366E, and R366E/F433S YidC proteins. The mutants were purified and Atto647N was attached to residue 405C. The fluorescently labeled YidC proteins were then reconstituted with 1,2-dioleoyl-sn-glycero-3-phosphocholine to generate proteoliposomes. After the purified Pf3 coat protein was labeled at residue 48 with the Atto520 dye in the cytoplasmic tail region, the Pf3 coat protein substrate was added to the proteoliposomes containing the YidC proteins in increasing amounts (see Experimental procedures). The binding efficiency of Pf3 coat protein to the YidC mutants was determined by FRET with a *K*_*d*_ of 241 ± 75 nM (SD of 10 independent measurements) for the WT and 349 nM ± 26 nM for R366E/F433S (experiments performed at room temperature). No *K*_*d*_ could be determined for the R366E mutant. Clearly, the R366E mutant was defective in the binding of the Pf3 coat protein, whereas WT YidC and the double mutant R366E/F433S were capable of efficient substrate binding.

We were able to identify a suppressor mutation (W234M) that rescued the activity of the *S. mutans* YidC2 (R73D) in which R73 corresponds to R366 in *E. coli* (22) (Fig. 5*A*). This *S. mutans* double mutant R73D/W234M had a mutation of the W234 residue corresponding to the *E. coli* Y517 YidC residue. Interestingly, when we made the corresponding Ser mutant in the *S. mutans* YidC2 R73D background, we found that this R73D/F151S YidC2 mutant (the *S. mutans* F151 residue aligns with F433 in *E. coli* YidC) was also fully functional at room temperature but inactive at 37 °C (Fig. 5*A*). Moreover, a mutant with a substitution of the nearby W234 residue, namely R73D/W234Y, was also found to be temperature-sensitive with little growth at 37 °C but functional at room temperature. The temperature-sensitive phenotype of these mutants suggests that the negatively charged residue in the groove destabilizes the protein at higher temperature. Figure 5, *B* and *C* summarizes the membrane insertion results of PC-Lep and Pf3-23Lep (also see Fig. S4) that is generally consistent with the complementation results. To our surprise, the insertion of PC-Lep with *S. mutans* R73D/W234M was inhibited, while Pf3-23Lep was almost completely inserted with the same YidC2 mutant. Possibly, this is due to the different insertion mechanisms used by the two substrates.

To explore the molecular mechanism that maintains the functionality of R366E/F433S YidC mutant, we performed a 100 ns simulation at 27 °C (Fig. 6*A*). Remarkably, the F433S suppressor mutation hydrogen bonds to the tyrosine 517 most of the time in the MD simulations (Fig. S6), which may stabilize the groove region. The electrostatic map computed from the simulation trajectory (Fig. 6*C*) predicts that the interior of the groove of the R366E/F433S mutant is indeed negatively charged (red color), while for the WT YidC (Fig. 6*B*), the groove is slightly positively charged (blue color).

We conclude from these results that YidC is functional with a negatively charged residue in the groove when there is a suppressor mutation, indicating that the electrostatic attraction between the arginine in the groove and the negatively charged residues in the substrate region is not required for YidC-mediated insertion.

## Discussion

The hydrophilic groove of all YidC family members possess a strictly conserved positively charged residue that has been proposed to interact electrostatically with the hydrophilic peptide chain of the substrate (18, 19). Evidence for this electrostatic attraction model comes from the finding that both the positively charged residue in the Gram-positive *B. subtilis* SpoIIIJ and the acidic residues in the N-tail of the substrate MifM are required for insertion (18). In addition, a positively charged residue in the groove is required for the insertase activity of the Gram-positive *S. mutans* YidC2 (24). However, the positive charge of this residue is not essential for the *E. coli* YidC or *A. thaliana* chloroplast Alb3 (24). This suggest that an electrostatic attraction mechanism due to the conserved arginine residue is not a general process required for substrate insertion into the groove prior to translocation.

Here, we show that the chemical nature of residue 517 (normally a tyrosine residue) of *E. coli* YidC determines whether the strictly conserved arginine is essential or not (Fig. 2). If an apolar residue is present at position 517, the arginine at 366 is essential, but when an aromatic or polar residue is present at 517, the arginine is no longer required (Fig. 3*C*). Similar results were observed for the Gram-positive *S*. *mutans* YidC2. By substituting W234 (analogous to *E. coli* Y517) with a more hydrophilic (serine or asparagine) residue, we were able to make the originally essential R73 dispensable (Fig. 3). Note that this *S. mutans* construct has amino acids 1 to 247 of the *E. coli* YidC fused to amino acids 25 to 310 of *S. mutans* YidC2. Fusing of *E. coli* YidC region possessing the nonconserved TM1 and large periplasmic region is necessary for correct membrane insertion and efficient activity of the *S. mutans* YidC2 in *E. coli* (24).

A closer look at the position of Y517 in the structure of *E. coli* YidC shows that it is located at the dome of the hydrophilic groove and is part of an aromatic cluster at the interface between the aqueous environment in the groove and the hydrophobic outer leaflet embedded portion of YidC. Interestingly, the water accessibility in the top one third of the groove is affected when Y517 is substituted with an apolar residue and is significantly decreased when the positive charge at 366 in the middle of the groove is simultaneously removed (Fig. 4, *A*–*C*). This was determined by examining the water accessibility in the groove by the reactivity of introduced cysteine residues to NEM and by EPR power saturation studies with a cysteine-incorporated spin label in the groove and monitoring whether the residue is lipid exposed or solvent exposed using paramagnetic O2 and NiEDDA quenchers, respectively. Finally, a decrease in the exposure of water surrounding the groove residues R366, S520, N521, and Q527 was corroborated by examining the number of waters in a fully equilibrated YidC in a lipid bilayer by MD simulations. We cannot rule out that the changes in solvation are caused by small conformational changes in the groove due to the interruption of the aromatic cluster. Alternatively, the water molecules may just fail to reach the top of the groove because of the additional hydrophobicity introduced with the hydrophobic sidechains at 517.

Taken together, our results suggest that the primary purpose of positively charged residue in the groove is to maintain the hydrophilic environment of the groove (Fig. 4), which is crucial for YidC function possibly by accommodating for the polar region of substrates during the process of insertion. This is in line with recent crosslinking results showing that the hydrophilic N-tail of the single-spanning Pf3 coat protein gets transiently incorporated into the hydrophilic groove and the C-region of N-tail interacts with the groove residues 517, 520, and 524 (23). The importance of hydrophilic microenvironment in the groove was also reported by Shimokawa-Chiba *et al.* (27) by substituting six polar residues in SpoIIIJ with Ala or Leu, both resulting in a defect in insertase activity.

Strikingly, YidC is functional when the strictly conserved arginine is replaced with a negatively charged residue (Fig. 5) with proper stabilization. We have found two suppressor mutations (F433S in *E. coli* YidC and W234M in *S. mutans* YidC2) that allow YidC to be functional with a negatively charged residue in the groove. In addition, *S. mutans* YidC2 variants with an aspartic acid substituting for R73 in combination with W234Y or F151S are fully active at room temperature, although impaired at 37 °C. Interestingly, in all cases, the suppressor mutations are part of the aromatic cluster, which we believe stabilizes the negatively charged residue in the YidC groove through indirect interactions. In the case of the R366E/F433S YidC mutant, MD simulations show that F433S forms a hydrogen bond to tyrosine 517 (Figs. 6*A* and S6). The combined results show negatively charged residues can function in the groove, suggesting that the electrostatic attraction is not generally required for YidC-mediated insertion.

We conclude from our biochemical, biophysical, and simulation studies that the hydrophilic characteristics of the membrane cavity surrounding the 366 residue are crucial to the protein translocation mechanism. This allows the hydrophilic region of the substrate to move into the membrane at least half way within a hydrophilic water-containing cavity (25). In addition, another important feature based on previous work is that YidC thins the bilayer in its vicinity (25). Therefore, this would reduce the energy cost of translocation since the distance by which a hydrophilic region would need to cross is reduced. Membrane thinning has been shown to operate in other transport systems such as BamA (28, 29), TatA (30), and the Hrd1 complex (for review see (26)). We propose that both features, membrane thinning and the presence of the hydrophilic membrane cavity, are mechanistically coupled in YidC family members to reduce the membrane barrier to promote translocation.

## Experimental procedures

### Materials, strains, and plasmids

Lysozyme, EDTA, L-(+)-Arabinose, D-(+)-Glucose, and Mal-PEG were purchased from Sigma-Aldrich. PEG6000 was purchased from Hampton Research. Proteinase K was from Qiagen. Isopropyl 1-thio-β-D-galactopyranoside was from Research Products International Corp. Phenylmethylsulphonyl fluoride was purchased from United States Biochemical (Affymetrix). Phosphate Buffered Saline, pH 7.2 and NEM were purchased from Thermo Scientific. Tran[^35^S]-label, a mixture of 85% [^35^S]-methionine and 15% [^35^S]-cysteine, 1000 Ci/mmol, was from PerkinElmer Life Sciences. Antiserum to leader peptidase (anti-Lep) was from our own laboratory collection. Anti-6X His tag antibody (horseradish peroxidase) was purchased from Abcam.

JS7131, the YidC depletion strain (from our collection), has its endogenous *yidC* inactivated and has a new copy of the *yidC* gene inserted at the lambda attachment site, under the control of the araBAD promoter (6). The low copy number plasmid pACYC184, which encodes chloramphenicol resistance, was used to express mutants of the *E. coli* YidC and the *S. mutans* 247YidC2 (31, 32). Expression of these pACYC184-encoded proteins is under control of the native *yidC* promoter in order to ensure close to chromosomal levels of protein expression. The 247YidC2 protein is comprised of residues 1 to 247 of the *E. coli* YidC fused to residues 25 to 310 of *S. mutans* YidC2 (31). The high copy number plasmid pMS119 (ampicillin resistant) was used to express Pf3-23Lep (33) or PC-Lep (34) under the control of the *lac* promoter. BL21(DE3) strain and pEH1 plasmid were used to express YidC single mutants in the Cys-alkylation assay. Mutator strain XL1-red (#200129) was purchased from Agilent for the randomized mutagenesis studies. All of the mutants of YidC homologs were constructed by site directed mutagenesis using PCR. All of the mutations were verified by DNA sequencing.

### Bacterial growth and pulse labeling

*E. coli* JS7131 was transformed with pMS119 encoding either Pf3-23Lep or PC-Lep and then further transformed with pACYC184-encoding YidC or 247YidC2. The cells were grown in LB media in the presence of ampicillin (final concentration, 100 μg/mL), chloramphenicol (final concentration, 25 μg/mL), and 0.2% arabinose. Depletion of the chromosomally encoded YidC was achieved by growth in the presence of 0.2% glucose for 4 h at 37 °C. The cells were subsequently washed twice with and resuspended in M9 medium +19 amino acids minus Met. After growing at 37 °C for another 30 min, expression of YidC-dependent membrane proteins was induced with 1 mM IPTG for 5 min, followed by pulse-labeling with [^35^S]-methionine (70 μCi/mL) for 1 min.

### Signal peptide processing and protease mapping

To analyze the membrane insertion of PC-Lep, a signal peptide cleavage assay was used. After [^35^S]-labeling at 37 °C, an equal volume of 20% TCA was added to precipitate total proteins. The precipitate was washed with ice-cold acetone and resuspended in Tris–SDS (pH 8.0) buffer. The samples were then subjected to immunoprecipitation with antiserum against Lep to examine membrane insertion by signal peptide processing of PC-Lep to coat-Lep.

Protease mapping was employed to assay membrane insertion of Pf3-23Lep. Following radiolabeling, the cells were collected by centrifugation at 4 °C and resuspended in 33 mM Tris–HCl (pH 8.0) with 40% sucrose. To prepare spheroplasts, lysozyme (5 μg/mL) and 1 mM EDTA (pH 8.0) were added and the sample was kept on ice for 30 min. Where indicated, PK was added (0.5 mg/ml final concentration) and incubated on ice for 1 h, followed by the addition of PMSF to quench the PK reaction. Immunoprecipitation of Pf3-23Lep was performed using Lep antiserum, and the samples were analyzed by SDS-PAGE and phosphorimaging.

The translocation efficiencies were determined by quantitation of the appropriate bands using Image J, developed at NIH. The percent of translocation across the membrane was determined as previously described (24). The Pf3-23Lep study was done in triplicates while the PC-Lep were done in duplicates.

### Complementation and Western blot assays

*E. coli* JS7131 cells bearing the respective pACYC184 plasmids were grown overnight at 37 °C in LB medium with 0.2% arabinose and 25 μg/mL chloramphenicol. In the following morning, the cells were washed once and back-diluted 1:100 into LB medium lacking arabinose. The cells were grown for 3 h at 37 °C and subsequently serially diluted (1:10, 1:100, 1:1000, and 1:10,000). Aliquots of each dilution (4 μL) were spotted on LB plates containing chloramphenicol and 0.2% arabinose or 0.2% glucose, respectively. The plates were incubated overnight at 37 °C.

The same overnight culture of JS7131, bearing pACYC184 encoding YidC or 247YidC2, was washed twice with and resuspended in LB containing glucose and chloramphenicol. After 4 h of growing at 37 °C, 600 μL of cells were precipitated with same volume of 20% TCA on ice for 1 h, washed with ice-cold acetone, and solubilized in Tris–SDS (pH 8.0). The samples were analyzed by Western blot with polyclonal antiserum against C-terminal peptides of *E. coli* YidC or *S. mutans* YidC2. YidC antibody was from our lab collection, while the antibody against *S. mutans* YidC2 was a gift from Jeanine Brady (University of Florida).

### Cys-alkylation assay

Cys-alkylation assays were performed as previously described (25) and were done in duplicates. An overnight culture of BL21(DE3) bearing pEH1-YidC single-Cys mutant was back-diluted 1:100 into fresh LB medium containing kanamycin (50 μg/mL) and grown to mid-log phase (A_600_ ≈ 0.55). One millimolar IPTG was added to induce YidC mutants for 30 min. The cells were subsequently washed twice with PBS, and the cell density was adjusted to A_600_ ≈ 2.0. Three aliquots (0.2 mL each) were made and the first aliquot was treated with NEM (0.5 mM final concentration) while the second and third were left untreated. These aliquots were then incubated at 30 °C for 30 min and subsequently washed twice with PBS buffer (with 5 mM DTT) and three times with PBS buffer (without DTT). To remove YidC aggregates, the cells were sonicated with Sonic Dismembrator Model 500 (Fisher Scientific) (microtip, 40% duty, eight cycles) while cooling. Intact cells and aggregates were removed by centrifugation at 16,000 g for 30 min at 4 °C. Supernatants were collected and precipitated with 10% (final concentration) ice-cold TCA for 1 h on ice. The pellets were washed with ice-cold acetone and solubilized with 100 μl Tris–SDS–Urea buffer (15 mM Tris–HCl, 6 M Urea, 2% SDS, pH 7.5). The first and third aliquots were treated with Mal-PEG (MW ∼ 5000 Da, 5 mM final concentration) for 40 min at 37 °C, while aliquot 2 was mock-treated with 5 mM PEG6000. The protein samples were then analyzed by Western blot using anti-6X His tag antibody (horseradish peroxidase).

### Randomized mutagenesis

A pACYC184 plasmid encoding *E. coli* YidC R366E mutant or *S. mutans* YidC2 R73D mutant was transformed into XL1-red and grown on LB plate with chloramphenicol overnight at 37 °C. The colonies were pooled and plasmids were isolated. The mixture of plasmids was then transformed into JS7131 and screened for active mutants on LB plates containing chloramphenicol and 0.2% glucose. The plates were incubated at 37 °C for 2 days. Plasmid DNA was extracted from picked colonies and analyzed by sequencing.

### Overexpression and purification of YidC

The overexpression and purification of pEH1-expressing YidC was carried out in *E. coli* BL21 cells. Individual colonies were picked and placed in 5 mL cultures for overnight growth in 50 μg/mL Kanamycin antibiotic. Overnight culture was used to inoculate 1 L LB culture and was grown at 37 °C until it reached A_600_ ≈ 0.6, at which point protein expression was induced using 1 mM IPTG. Cells were harvested 3 h postinduction by centrifugation (at 3200*g* for 20 min, 4 °C) and stored at −80 °C.

Cell pellets were thawed on ice and resuspended in PBS buffer pH 7.2 containing Lysozyme (1 mg/mL). The cells were sonicated on ice for several cycles at an output of 65% power to break open the membranes. The samples were centrifuged at 40,000*g* for 50 min at 4 °C to remove unlysed cells and inclusion bodies. To isolate the membrane pellets, the supernatant was then subjected to ultracentrifugation at 160,000*g* for 50 min at 4 °C. Membrane pellets were then solubilized in PBS containing 1% n-Dodecyl-beta-D-Maltoside (DDM) (Anatrace) overnight by stirring with magnetic beads. The sample was again centrifuged for 25 min to remove all nonsolubilized components, and the supernatant was incubated with the Co^2+^-NTA matrix (Qiagen) for 3 h at 4 °C. After washing with low imidazole buffer [PBS (pH 7.2), 20 mM imidazole, 10% glycerol, and 0.2% (w/v) DDM], 3 mg of the spin label MTSL was added to the YidC sample on the column and shaken for 24 h at 4 °C. YidC was eluted from the column with high imidazole buffer [PBS (pH 7.2), 400 mM imidazole containing 0.2% (w/v) DDM] and collected in 1 mL fractions. The samples were analyzed by 15% SDS-PAGE and the fractions containing pure YidC were pooled and dialyzed with buffer [PBS pH 7.2, 0.02% (w/v) DDM] overnight to remove glycerol, imidazole, and any free spin label. The YidC protein was concentrated by centrifugation using a 50 kDa Amicon spin concentrator.

### Reconstitution of YidC into proteoliposomes

1,2-Dioleoyl-sn-glycero-3-phosphocholine was purchased from Avanti Polar Lipids (Alabaster) and lipid vesicles prepared. The dry lipid film was resuspended in HEPES buffer (pH 8.0) containing 50 mM KCl, 0.02% (w/v) DDM, and incubated at 37 °C for 1 h. The sample was vortexed at a low speed to form multilamellar vesicles. Unilamellar vesicles were generated by the extrusion technique (Mini-Extruder, Avanti Polar Lipids Inc). Specifically, 1 mL of the lipid suspension was extruded 7 to 11 times through a membrane with a pore size of 0.4 μm until a semi-clear solution is achieved. For preparing proteoliposomes, a concentrated spin-labeled YidC sample (at a final concentration of 20 μM) was added to the lipid sample and extruded as described above. The final protein:lipid molar ratio was set to 1:400. SM2-Biobeads were prepared according to the manufacturers protocol and added to the proteoliposomes to remove excess DDM as described in Kusters *et al.* (35) The removal of DDM in the lipid sample with the Biobeads aids in further reconstitution and is achieved by slow rotary shaking of the sample overnight at 4 °C. The Biobeads are removed by centrifugation at 10,000 rpm for 5 min. The proteoliposomes are concentrated by centrifugation at 200,000*g* to form a pellet and resuspended in 15 μL of HEPES buffer for EPR measurements.

### Simulated systems and simulation parameters

A system with an equilibrated (∼185 ns), crystallography-based model of the WT YidC protein embedded in a lipid bilayer (75% POPE and 25% POPG) (25) was used as a starting conformation for the WT simulation. The same system was used along with the psfgen VMD (36) plugin to create three mutated versions of the equilibrated YidC and to neutralize the corresponding systems (Table 1). Molecular dynamics simulations using periodic boundary conditions were performed using NAMD 2.12 (37), the CHARMM36 force field for proteins and lipids with the CMAP correction and the TIP3P model for water (38, 39, 40). A cutoff of 12 Å with a force-based switching function starting at 10 Å was used for van der Waals interactions. A 2 fs integration time step was used together with SHAKE. The Particle Mesh Ewald method was used to compute long-range electrostatic forces every other time step without cutoff and with a grid point density of >1 Å^−3^. Langevin dynamics was utilized to enforce constant temperature *T* = 300 K with a damping coefficient of 0.1 ps^−1^. Constant pressure simulations (*NpT*) at 1 atm were conducted using the hybrid Nosé-Hoover Langevin piston method with a 200 fs decay period and a 100 fs damping time constant. Each system was energy-minimized and equilibrated in the *NpT* ensemble (Table 1). Harmonic constrains (*k*_*s*_ = 1 kcal mol^−1^ Å^−2^) to backbone atoms were applied when indicated. Coordinates of all atoms in the system were saved every 2 ps. Computation of the electrostatic potential was carried out using the VMD PMEpot plugin with an Ewald factor of 0.25 Å^−1^ using conformations saved every 80 ps for simulations in which YidC’s backbone was constrained. Molecular images were created with the molecular graphics program VMD (36).

**Table 1:** Summary of simulations

Label	System	*t*_sim_ (ns)	Type	Start	Constraints	Size (#atoms)	Initial size (nm^3^)
S1a	WT	100	EQ^a^	−	backbone	197,865	11.3 × 12.2 × 14.1
S1b		103	NpT	S1a	−		11.8 × 12.3 × 13.3
S2a	R366E	100	EQ^a^	−	backbone	197,852	11.3 × 12.2 × 14.1
S2b		109	NpT	S2a	−		11.2 × 12.6 × 13.6
S2c^b^		100	EQ^a^	S2a	backbone		11.2 × 12.6 × 13.6
S2d		82	NpT	S2c	−		11.3 × 12.8 × 13.4
S3a	R366E/F433S	100	EQ^a^	−	backbone	197,843	11.3 × 12.2 × 14.1
S3b		104	NpT	S3a	−		12.4 × 11.5 × 13.4
S4a	R366N/Y517I	100	EQ^a^	−	backbone	197,851	11.3 × 12.2 × 14.1
S4b		104	NpT	S4a	−		11.0 × 13.2 × 13.3
Total		1002					

a EQ indicates simulations that consisted of 1000 steps of minimization and 100 ns of dynamics with the protein backbone constrained (*k* = 1 kcal/mol/Å^2^) in the *NpT* ensemble (γ = 0.1 ps^−1^).

b A potassium ion found inside YidC was exchanged with a bulk water molecule before starting simulation S2c.

### CW power saturation experiments

Power saturation experiments were performed on a Bruker EMX X-band CW-EPR spectrometer consisting of an ER 041XG microwave bridge coupled with an ER 4123D CW-Resonator (Bruker BioSpin) (41). The samples were loaded into gas permeable TPX capillary tubes with a total volume of 4 to 6 μL at a spin label concentration of 40 to 80 μM. EPR data collection was carried out using a modulation amplitude of 1 G and a varying microwave power of 0.4 to 100 mW. The scan range of all spectra was 90 G, and the final spectra were obtained by signal averaging 10 scans.

CW-EPR power saturation curves were obtained for all the spin labeled YidC Cys mutants under three conditions: (1) equilibrated with nitrogen as a control; (2) equilibrated with a lipid-soluble paramagnetic reagent: air (20% oxygen); and (3) equilibrated with nitrogen in the presence of a water-soluble paramagnetic reagent NiEDDA chelate (2 mM), as described (41). The samples were purged with gas for at least 60 min at a rate of 10 mL/min before performing each EPR measurement. High purity nitrogen and house supply compressed air lines were used. The resonator remained connected to the gas line during all measurements, and the sample temperature was held at 295 K. The peak-to-peak amplitude (A) of the first derivative *m*_*I*_ = 0 resonance line was measured and plotted against the square root of the incident microwave power. The data points were then fit using a Matlab software script using Equation 1:

(1) $$A=I\sqrt{P}{\left[1+\left(2^{1/\varepsilon }-1\right){P/P}_{1/2}\right]}^{-\varepsilon }$$

Where *I* is a scaling factor, *P*_1/2_ is the power where the first derivative amplitude is reduced to half of its unsaturated value, and *ε* is a measure of the homogeneity of saturation of the resonance line. In the above equation, *A*, *I*, *ε*, and *P* are adjustable parameters and yield a characteristic *P*_1/2_ value. The corresponding *Φ* depth parameters were calculated using the following equation:

(2) $$\Phi =ln\left(\frac{\Delta P_{1/2}\left({\text{O}}_{2}\right)}{\Delta P_{1/2}\left(\text{NiEDDA}\right)}\right)$$

Where Δ*P*_1/2_(O2) is the difference in the P_1/2_ values for air- and nitrogen-exposed samples, and Δ*P*_1/2_(Ni-EDDA) is the difference in the P_1/2_ values for NiEDDA and nitrogen-exposed samples (41).

### Substrate binding to YidC by a FRET assay

A cysteine was first incorporated at position 405 of a cysteine-less YidC at the cytoplasmic face of the membrane into the WT and the mutants 366E and 366E/433S. The 405C residues of the YidC mutants were used to attach a fluorescent label with the maleimide dye Atto520 (donor). Residue 48 of the substrate Pf3 was labeled with Atto647N (acceptor). For the labeling reaction, 1.3 fold excess of the dye was added to the protein and incubated for 1.5 h at room temperature. The free dye was separated from the protein by size-exclusion chromatography with a Superdex 200 10/30 column, and the YidC proteins were reconstituted in 1,2-dioleoyl-sn-glycero-3-phosphocholine liposomes. The fluorescence measurements to examine binding were performed on a Fluorolog 2.3 (Jobin Yvon-Spex). The extinction wavelength was set at 500 nm to excite the donor dye (Atto520), and the emission wavelength was scanned from 510 to 700 nm. The fluorescence signal of the acceptor was monitored at 669 nm. The Atto647N-Pf3 coat protein was added in steps, each after 1 min, to the proteoliposomes containing 300 nM Atto520-YidC. The concentrations in each of the steps were 60, 60, 150, 150, 150, 300, and 300 nM, reaching a final concentration of 1.17 μM. The measurement started after an incubation period of 1 min after the addition of the Pf3 coat substrate. For our analysis of the binding of Pf3 coat to YidC, we plot the ΔF_Acceptor_ signal:

(3) $$\Delta F_{Acceptor}=\;\left|F_{A_{i}}-F_{A_{0}}\right|$$

$F_{A_{0}}$ is the initial fluorescence values at 669 nm of YidC without labeled Pf3, and $F_{A_{i}}$ is the value of the fluorescence signal at the i-th titration step of labeled Pf3.

The measurements were background-corrected by a buffer and labeled Pf3 only spectrum. The values of the fluorescence signal were corrected by a titration factor of the added Pf3.

For the calculation of the *K*_*D*_, the binding curve was fitted against the following equation:

(4) $$\Delta F=\;\Delta F_{max}\;\frac{\left[Pf3\right]}{\left[Pf3\right]+\;K_{D}}$$

(5) $$K_{D}=\frac{\Delta F_{max}*\left[Pf\;3\right]}{\Delta F}-\left[Pf3\right]\;$$

Where $\Delta F_{max}$ is the maximum fluorescence signal change reached at saturation.

## Data availability

All data generated for this study are included within this article.

## Supporting information

This article contains supporting information.

## Conflict of interest

The authors declare that they have no conflicts of interest with the contents of this article.
