# Supplementary material for: A hydrophilic microenvironment in the substrate-translocating groove of the YidC membrane insertase is essential for enzyme function
Source: J Biol Chem. 2022 Feb 9;298(3):101690. doi: 10.1016/j.jbc.2022.101690 (PMC8920935; doi:10.1016/j.jbc.2022.101690)
Supplement: Supplemental Figures S1–S4 [file mmc1.docx]

**The hydrophilic microenvironment in the substrate-translocating groove of the YidC membrane insertase is essential for enzyme function**

Yuanyuan Chen^1^, Marcos Sotomayor^1^, Sara Capponi^2,3^, Balasubramani Hariharan^1^, Indra D. Sahu^4,7^ Maximilian Haase^5^, Gary A. Lorigan^4^, Andreas Kuhn^5^, Stephen H. White^6^, and Ross E. Dalbey


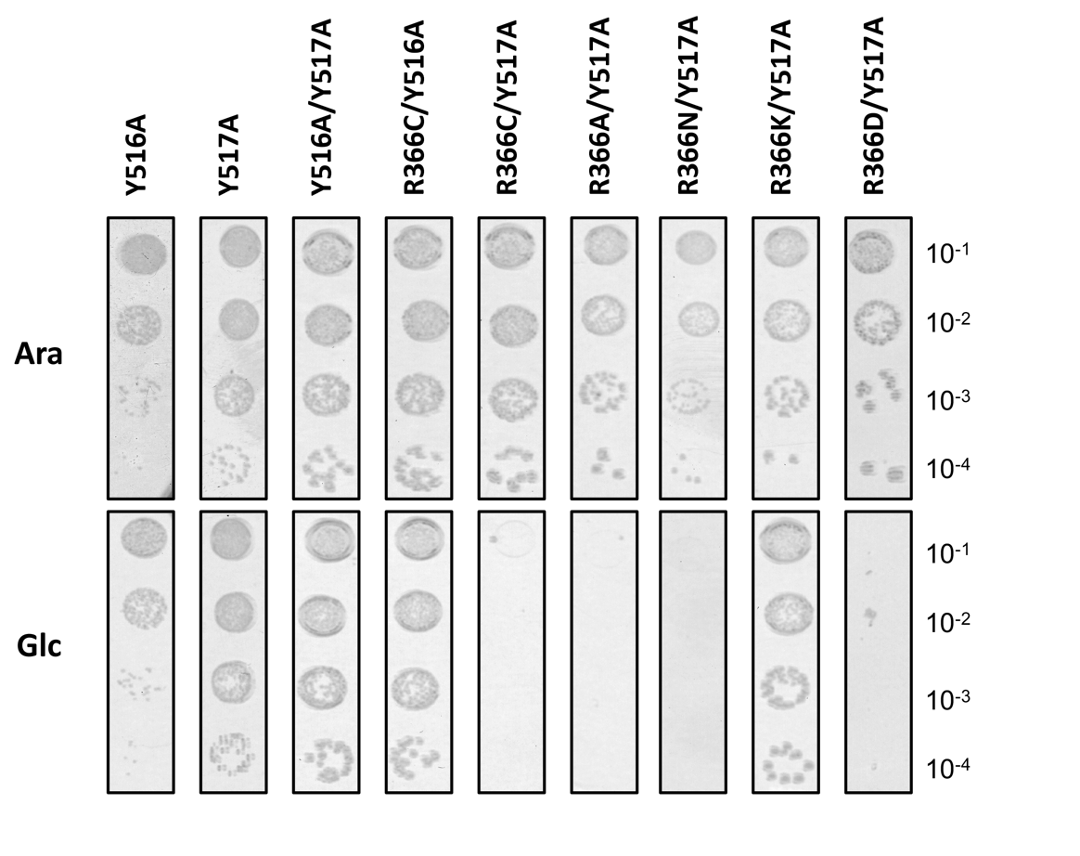


**Figure S1. The *E. coli* YidC Y517A mutant requires a positive charge in the hydrophilic groove for function.** Complementation assay was performed to examine the importance of the R366 residue for *E. coli* YidC function when Y517 was substituted with Ala. The R366 residue was mutated to Ala, Cys, Asn, Lys or Asp in pACYC184 in combination with the Y517A mutation, and transformed into JS7131. Y516 was also substituted with Ala, or both Y516 and Y517 were changed to Ala, Y516 was mutated to Ala in combination with R366 mutated to Cys and Y517 in combination with R366 Asn, Lys and Asp, respectively. The spot tests were performed as described in Fig. 2A. Note that the data for R366A/Y517A and Y517A (see Fig. 2A) is included here for comparison.


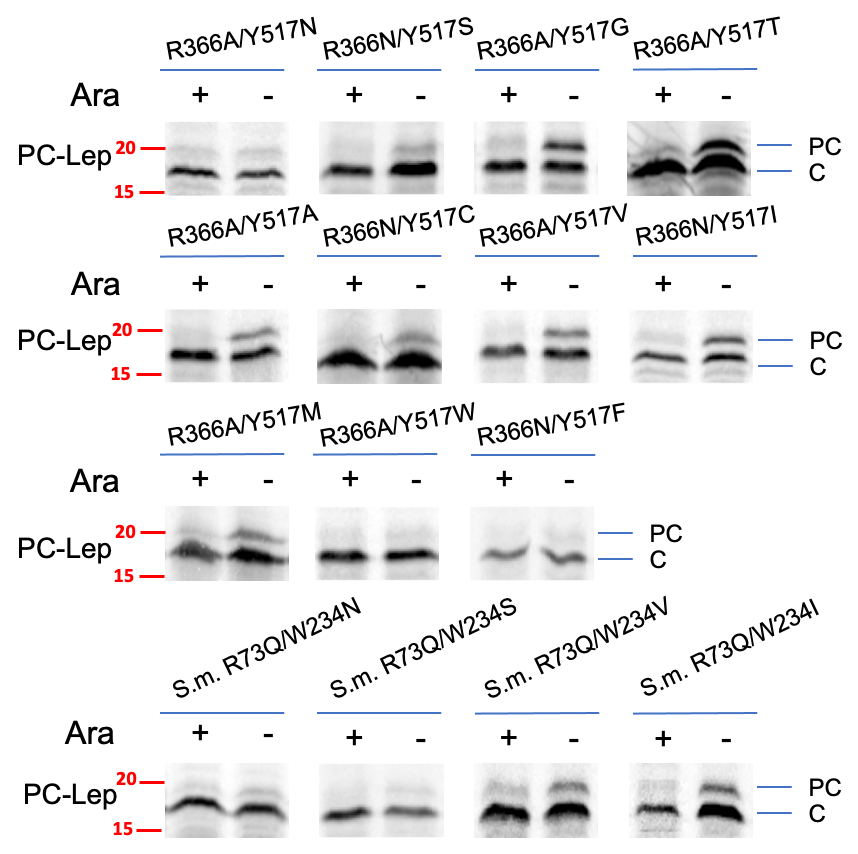


**Figure S2A. Detailed results of insertion assay performed for Fig. 3D. A.** Signal peptide processing assay was used to examine the insertion efficiency of PC-Lep as described in Fig. 2B. Red numbers indicate molecular weight marker values in kDa.


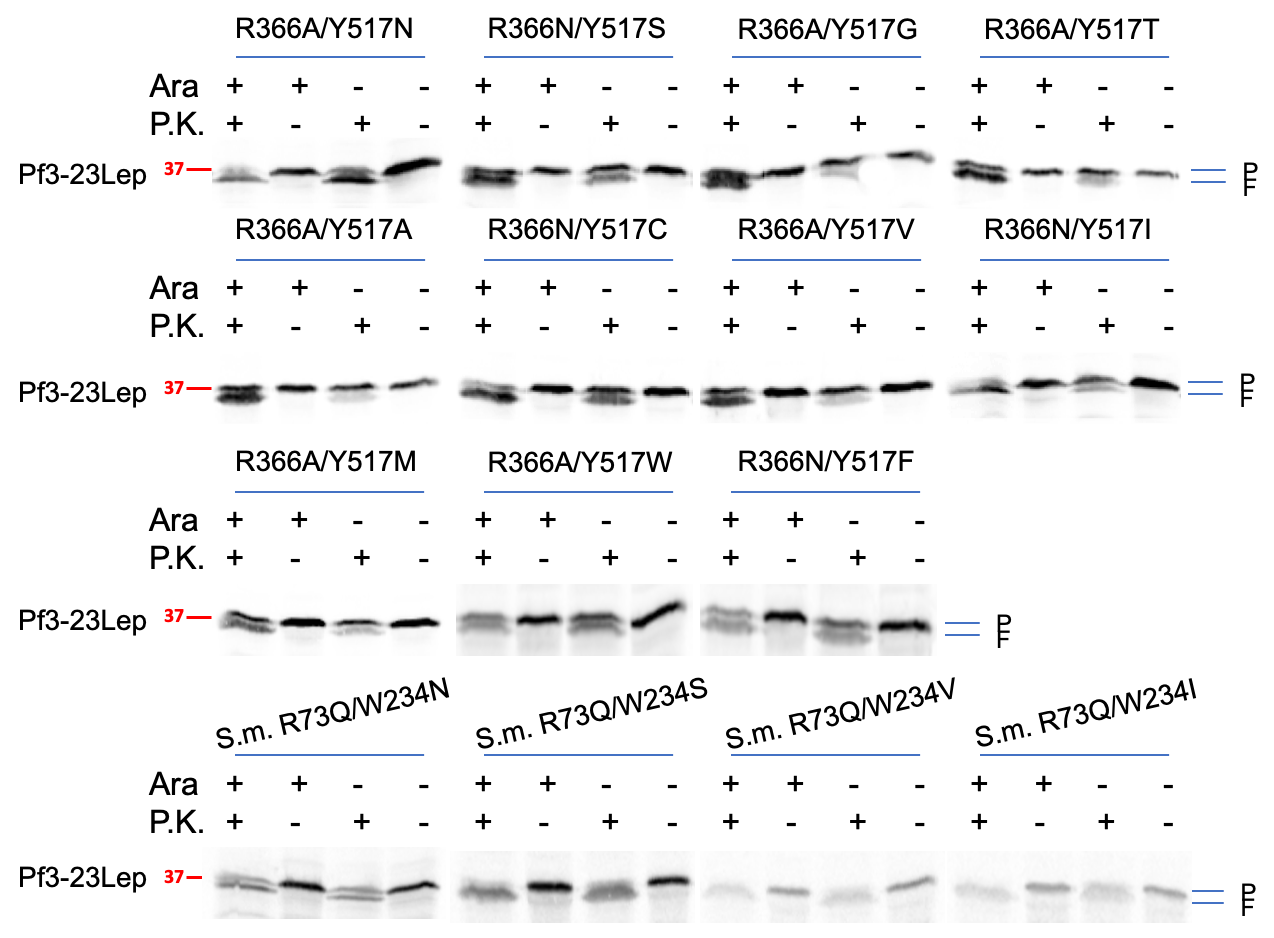


**Figure S2B. Detailed results of insertion assay performed for Fig. 3D. B.** The capability of *E. coli* YidC and *S. mutans* YidC2 mutants to insert Pf3-23Lep was tested by protease mapping assay as described in Fig. 2C. Red numbers indicate molecular weight marker values in kDa.


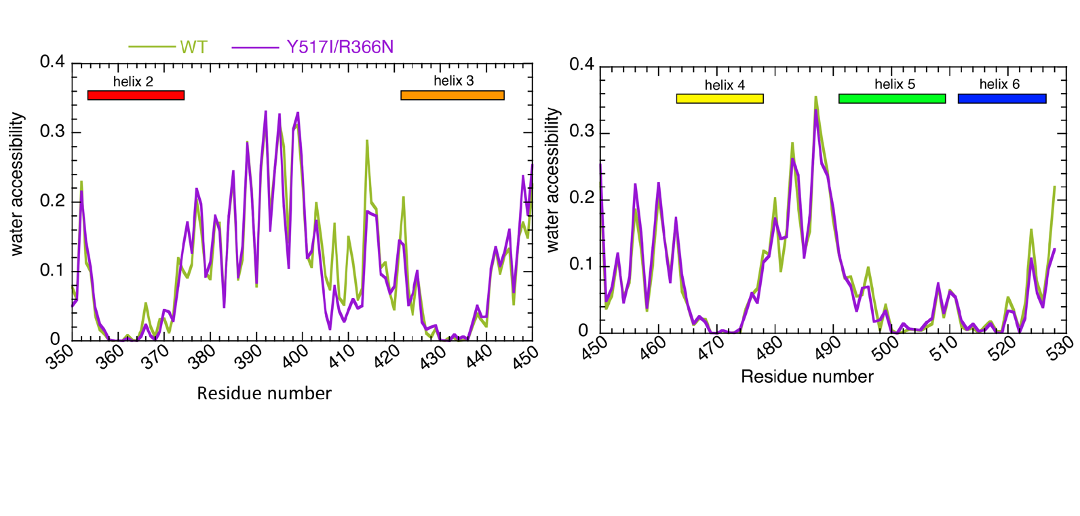


**Figure S3. The water accessibilities of *E. coli* YidC wild-type and Y517I/R366N mutant determined from the MD simulations of YidC in POPE/POPG bilayers.** A. Accessibility results from residues 350-450. B. Accessibility results for residues 450-528.


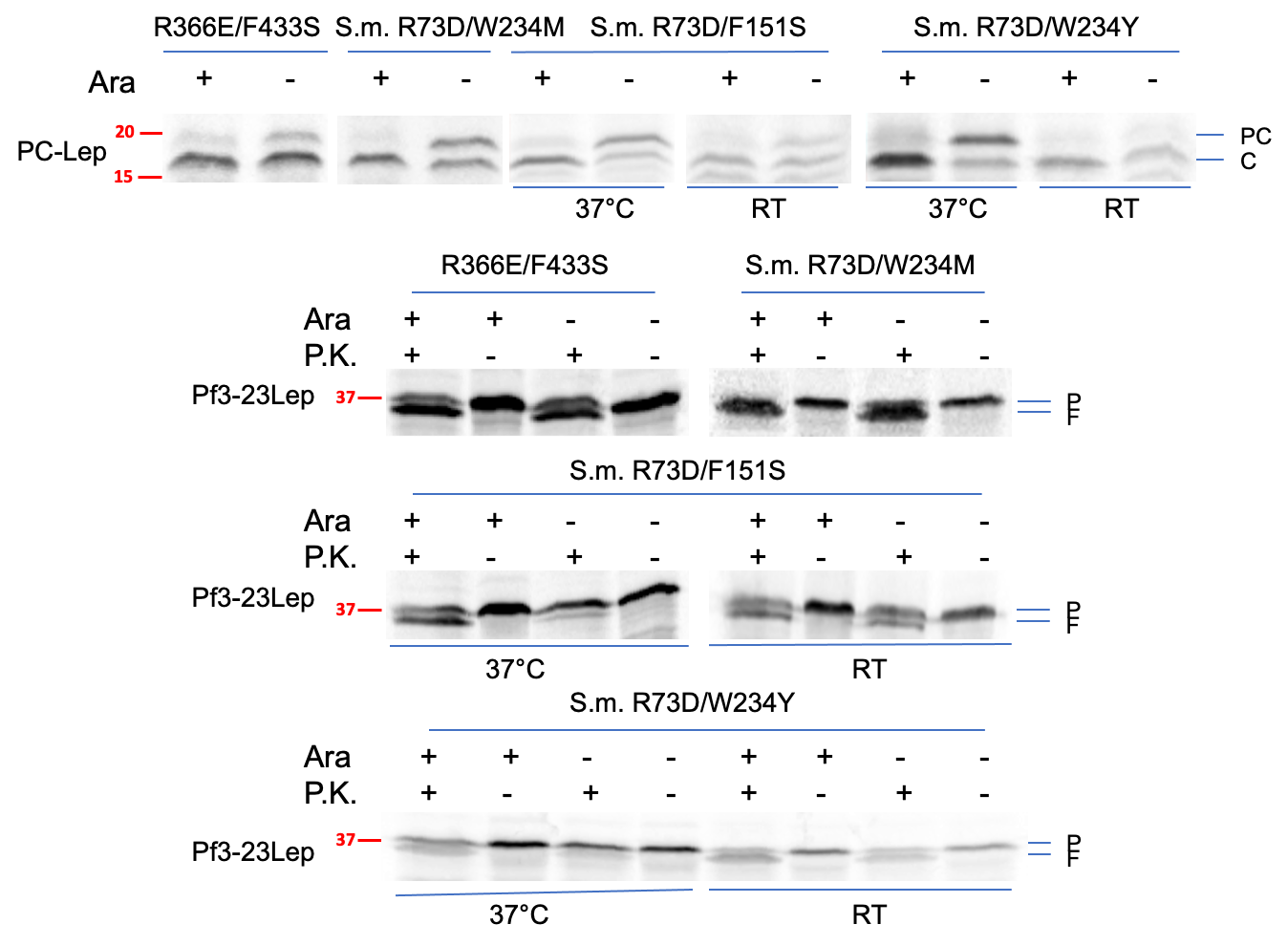


**Figure S4. Detailed results of insertion assay with the negatively charged YidC mutants performed for Fig. 5B.** Signal peptide processing (**A**) and protease mapping (**B**) assays were applied to examine the insertase activity of *E. coli* YidC and *S. mutans* YidC2 negative mutants for PC-Lep (**A**) and Pf3-23Lep (**B**), respectively. Red numbers indicate molecular weight marker values in kDa.


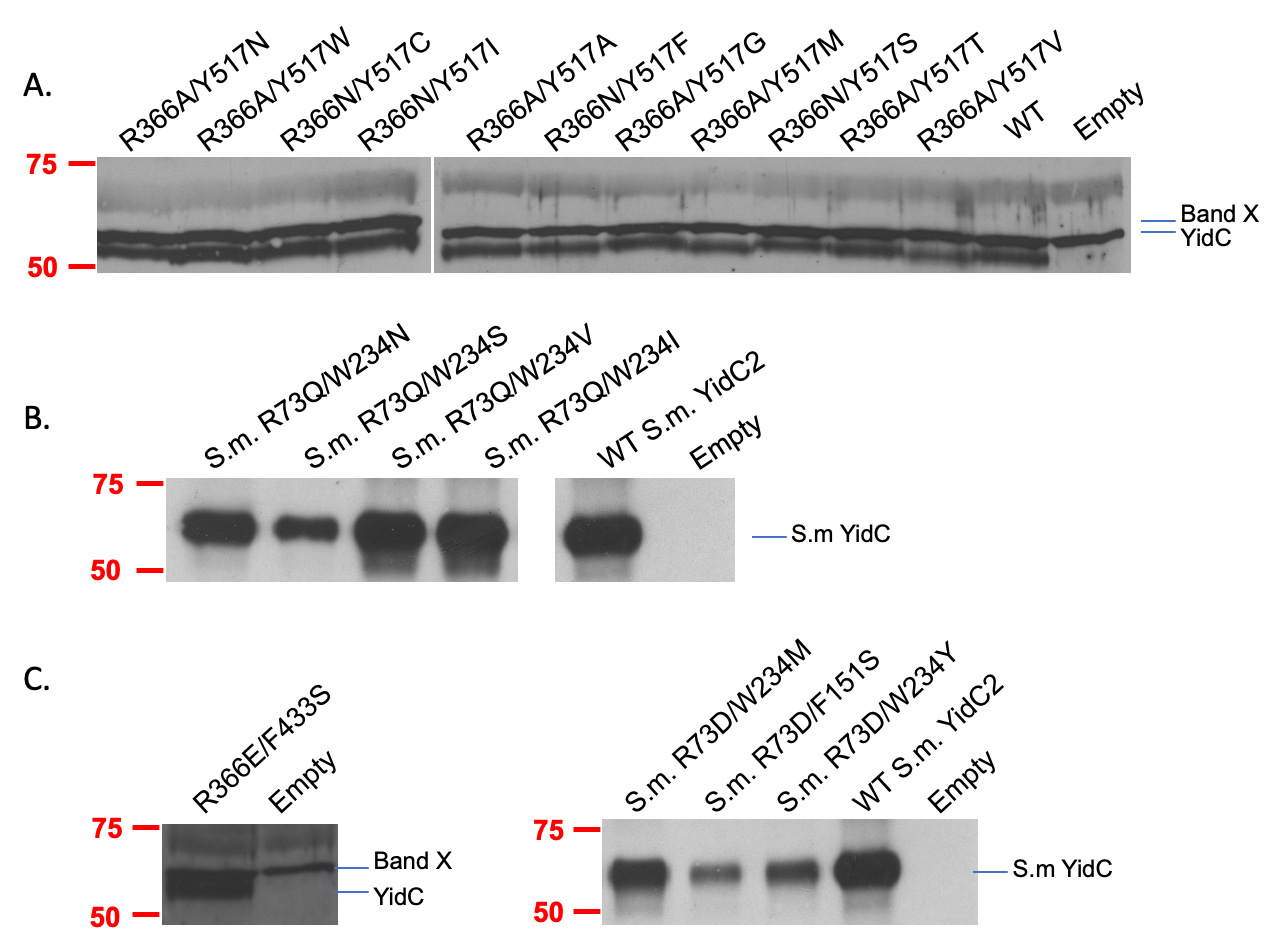


**Figure S5. Western blots to check for expression levels of *E. coli* YidC and *S.* *mutans* YidC2 mutants.** (**A-B**) Expression of *E. coli* YidC mutants (**A)** and *S. mutans* YidC2 mutants (**B**) for the studies in Fig. 3D. (**C**) Expression of the negatively charged *E. coli* YidC (left) and *S. mutans* YidC2 mutants (right) shown in Fig. S5B. In the panel for the *E. coli* YidC mutants, the top band is an unrelated protein recognized by our antiserum. The last two lanes in Fig. S5B are also used on the right-side of Fig. S5C as comparison controls. Red numbers indicate molecular weight marker values in kDa.

**
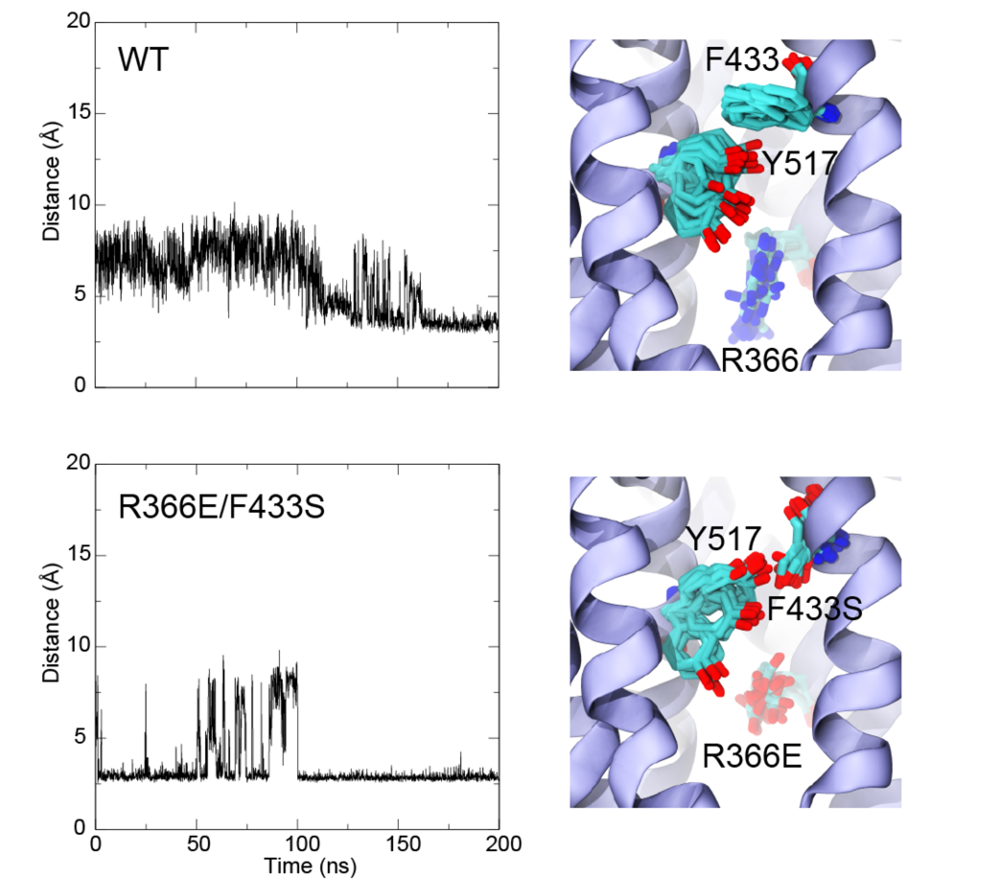
**

**Figure S6. Dynamics of YidC residues controlling substrate translocation.** Distance between atoms F433-Cγ and Y517-O (top left) and atoms F433S-Oγ and Y517-O (bottom left) versus time during simulations S1a-b and S3a-b, respectively. Right panels show detail of YidC’s hydrophilic groove with key residues shown in stick representation for superposed conformations taken every 8 ns of simulation trajectory. Side chains of residues F433S and Y517.
